# Phytohormone treatment induces generation of cryptic peptides with antimicrobial activity in the Moss *Physcomitrella patens*

**DOI:** 10.1186/s12870-018-1611-z

**Published:** 2019-01-07

**Authors:** Igor Fesenko, Regina Azarkina, Ilya Kirov, Andrei Kniazev, Anna Filippova, Ekaterina Grafskaia, Vassili Lazarev, Victor Zgoda, Ivan Butenko, Olga Bukato, Irina Lyapina, Dmitry Nazarenko, Sergey Elansky, Anna Mamaeva, Vadim Ivanov, Vadim Govorun

**Affiliations:** 1 Laboratory of Proteomics, Shemyakin and Ovchinnikov Institute of Bioorganic Chemistry, Russian Academy of Sciences, Moscow, Russia; 2 Federal Research and Clinical Center of Physical-Chemical Medicine of Federal Medical Biological Agency, Moscow, Russia; 3 Moscow Institute of Physics and Technology, Dolgoprudny, Moscow region Russia; 4 Institute of Biomedical Chemistry, Moscow, Russia; 5 Department of Analytical Chemistry, Faculty of Chemistry, Lomonosov Moscow State University, Moscow, Russia; 6 Biological Faculty, Lomonosov Moscow State University, Moscow, Russia

**Keywords:** LC-MS/MS, Peptidome, Plant immunity, *Physcomitrella patens*, Secretome, Phytohormones

## Abstract

**Background:**

Cryptic peptides (cryptides) are small bioactive molecules generated via degradation of functionally active proteins. Only a few examples of plant cryptides playing an important role in plant defense have been reported to date, hence our knowledge about cryptic signals hidden in protein structure remains very limited. Moreover, little is known about how stress conditions influence the size of endogenous peptide pools, and which of these peptides themselves have biological functions is currently unclear.

**Results:**

Here, we used mass spectrometry to comprehensively analyze the endogenous peptide pools generated from functionally active proteins inside the cell and in the secretome from the model plant *Physcomitrella patens*. Overall, we identified approximately 4,000 intracellular and approximately 500 secreted peptides. We found that the secretome and cellular peptidomes did not show significant overlap and that respective protein precursors have very different protein degradation patterns. We showed that treatment with the plant stress hormone methyl jasmonate induced specific proteolysis of new functional proteins and the release of bioactive peptides having an antimicrobial activity and capable to elicit the expression of plant defense genes. Finally, we showed that the inhibition of protease activity during methyl jasmonate treatment decreased the secretome antimicrobial potential, suggesting an important role of peptides released from proteins in immune response.

**Conclusions:**

Using mass-spectrometry, *in vitro* experiments and bioinformatics analysis, we found that methyl jasmonate acid induces significant changes in the peptide pools and that some of the resulting peptides possess antimicrobial and regulatory activities. Moreover, our study provides a list of peptides for further study of potential plant cryptides.

**Electronic supplementary material:**

The online version of this article (10.1186/s12870-018-1611-z) contains supplementary material, which is available to authorized users.

## Background

Peptides play an important role in growth and development, stress-induced responses and defense mechanisms [1–4]. As such, the peptidome can be considered the “dark matter” of the proteome: it includes thousands of potentially active molecules whose functions are not yet fully understood. Most bioactive plant peptides analyzed to date are generated via processing of inactive protein precursors. However, recent studies have shown that bioactive peptides can also be directly translated from short open reading frames [1, 5, 6]. In addition, a substantial part of plant cell peptidome comprises the proteolytic degradome, i.e., peptides generated via degradation of functional proteins [7].

While degradome peptides are often ignored as merely breakdown products resulting from regulation of active proteins, there is increasing evidence that peptides of the proteolytic degradome can be biologically functional. A number of cryptides, bioactive peptides hidden in protein structures, have been described in human [8–10]. Among plant cryptides, inceptin is a fragment of chloroplast ATP-synthase from Chinese cowpea (*Vigna unguiculata*), GmSubPep and GmPep914 are fragments of subtilisin-like protease in soybean (*Glycine max*) and CAPE-1 is a fragment of an active PR-1 tomato protein [11–14]. Each of these plant peptides plays a role in plant defense as an elicitor molecule.

Of special interest are bioactive peptides generated from functional proteins under stress conditions or hormone treatment. Hormone signaling in response to pathogen invasion can lead to the activation of protective mechanisms in plants. Phytohormones induce the proteolysis of certain proteins, thereby influencing the peptidome landscape [14–17]. This proteolysis has also been reported to generate peptides with biological activity. For instance, methyl jasmonate (MeJA) causes degradation of the tomato PR-1 protein and secretion of the peptide CAPE1, which regulates plant defense responses and has insecticidal activities [14, 15]. These results indicate that the degradome peptides represent more than simply a reflection of the breakdown of the proteome. However, although the effects of hormones on the intact proteome have been catalogued in several tissues, species and conditions, the converse impact of stress hormones on peptidogenesis itself in plant cells has not yet been assessed [18, 19]. Some peptides are secreted into the extracellular space, contributing to the plant cell secretome [20, 21]. For instance, defensins are secreted plant peptides that have antimicrobial activity [22–26]. Only a few secreted plant peptides have been well studied, such as CLE (CLAVATA3 [CLV3]/ENDOSPERM SURROUNDING REGION [ESR]), CRP (cysteine-rich) and PSK (phytosulfokine) [27–35]. Since no comprehensive analysis of peptides in the plant secretome has been conducted to date, our knowledge of their functions and origins remains limited.

We previously performed a systematic study of peptidogenesis in gametophores, protonemata and protoplasts of *P. patens* and found that the composition of the peptide pools (a set of all identified endogenous peptides) strongly differs between these cell types [7]. According to the recent studies, moss secretome contains a number of proteins, including different types of proteases and stress-responsive proteins [36, 37]. However, their role in the extracellular peptide pools formation remains to be elucidated. The aim of the present study was to carry out comprehensive analysis of the peptides of the *P. patens* secretome and to examine the influence of stress hormones on the cell peptidome and secretome. Using mass-spectrometry analysis, we analyzed pools of native peptides extracted from cells and the culture medium of the moss *P. patens* before and after treatment with the phytohormone MeJA. Hormone treatment led to a dramatic increase in the sizes of peptide pools both within the cell and in the secretome. We also observed an increase in the proteolysis rate of precursors under MeJA treatment, as well as hormone-specific degradation of new functional proteins. Moreover, the protein degradation patterns significantly differed between the cell and the secretome, suggesting that distinct types of proteolytic enzymes might be located in these compartments. Using *in silico* and *in vivo* analysis of the identified peptides from functionally active proteins upon treatment with MeJA, we detected antimicrobial activity in a number of the generated peptides. We identified the cryptic peptide, which has the highest predicted antimicrobial potential in the secretome, influenced the transcription of pathogenesis-related genes in a time-dependent manner. Therefore, the plant peptidomes include a number of bioactive peptides originating from functional proteins whose roles were previously underestimated.

## Results

### Methyl jasmonate induces changes in both intracellular and secretome peptidomes

*Physcomitrella patens* responds to MeJA exogenous treatment by growth inhibition and induction of pathogenesis-related genes [38–40]. To determine whether this stress hormone influences the plant peptidome, we identified intracellular and extracellular endogenous peptides in the control and MeJA-treated moss protonema cells and secretome samples (Additional file 1: Figure S1). To check if the process of sample preparation influences the peptidome composition, we added eleven synthetic peptides (RT peptides), having a broad range of retention time to original samples (cell homogenate or culture media in case of secretome). All RT peptides were then identified both in the secretome and the cell samples by Multiple Reaction Monitoring (MRM) experiment, demonstrating a similar peptide recovery in both extraction procedures (Additional file 2: Figure S2, Additional file 3: Table S1).

**Fig. 1:**
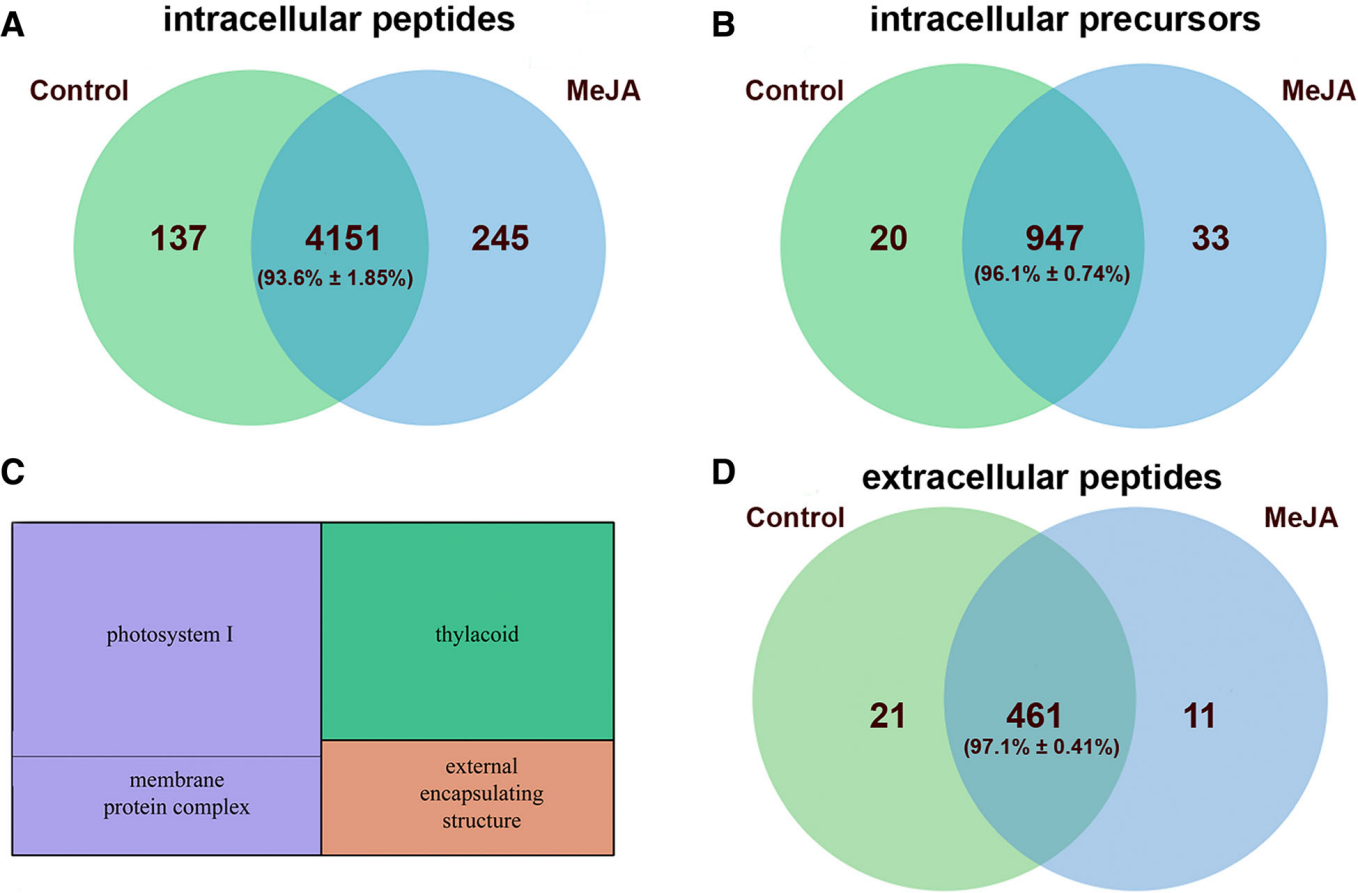
The comparison among endogenous peptides and their precursors between control and MeJA-treated samples. Venn diagram showing a comparison among endogenous peptides (**a**) and their precursors (**b**) between control and MeJA-treated protonema cells (*n*=3 independent biological repeats). **c** TreeMap showing GO enrichment terms (“Cellular component”) in a set of secretome peptide precursor proteins (*p*-value<0.05). The size of each square corresponds to the -log10(*P*-value). **d** Venn diagram showing a comparison among secreted peptides from protonemata treated with MeJA and control samples (*n*=3 independent biological repeats). The mean percentage with standard deviation for common peptides is shown

We examined endogenous peptides extracted from *P. patens* protonema cells treated with 0.4 mM MeJA. Taking into account the heterogeneity of peptide pools, for further analysis peptides identified in at least two biological repeats were selected. Overall, 4533 endogenous peptides belonging to 1000 protein precursors have been identified (Fig. 1a, b). We identified about 45 % peptides as C-terminal peptides, defined as starting within 50 amino acid of the C-terminus of the protein. While a significant portion of the peptide pool was identical between control and treated cells, MeJA resulted in the generation of 245 (about 5.5 % of total peptides) additional peptides; 67 (27 %) of these peptides were generated from new precursors in MeJA-treated cells (Additional file 4: Table S2). The distribution of peptide intensities showed a high correlation between MeJA-treated and control sample (Pearson r=0.68, *p*-value 5.4e-284; Additional file 5: Figure S3). Most peptides in the cell peptidome originated from proteins involved in photosynthesis, the Calvin cycle, glycolysis and sucrose biosynthesis (Additional file 6: Figure S4). MeJA-treated samples had more identified peptides per protein compared with the control.

We then used mass-spectrometry analysis to identify peptides in the protonema culture medium and to explore the impact of MeJA on the cell secretome. We detected 482 peptides in protonema culture medium derived from 114 proteins in at least two biological repeats (Additional file 7: Table S3). According to GO analysis, most of the precursors represent photosystem I and extracellular or extrinsic membrane proteins, such as alpha-expansin (EXPA6), pectin methylesterase and others (Fig. 1c). We identified 11 additional unique peptides after short-term treatment (1 h) of the protonemata with 0.4 mM MeJA (Fig. 1d). The precursors of plant bioactive peptides and hormones are often small proteins without predicted functions [1]. In the moss secretome, we also identified peptides originated from thirty-nine small (<200 aa) proteins. Three of them were predicted proteins, which gave rise to peptides whose abundance, increased upon MeJA treatment. The possible function of these peptides remains to be elucidated.

### Changes in protein degradation patterns under MeJA treatment

**Fig. 2:**
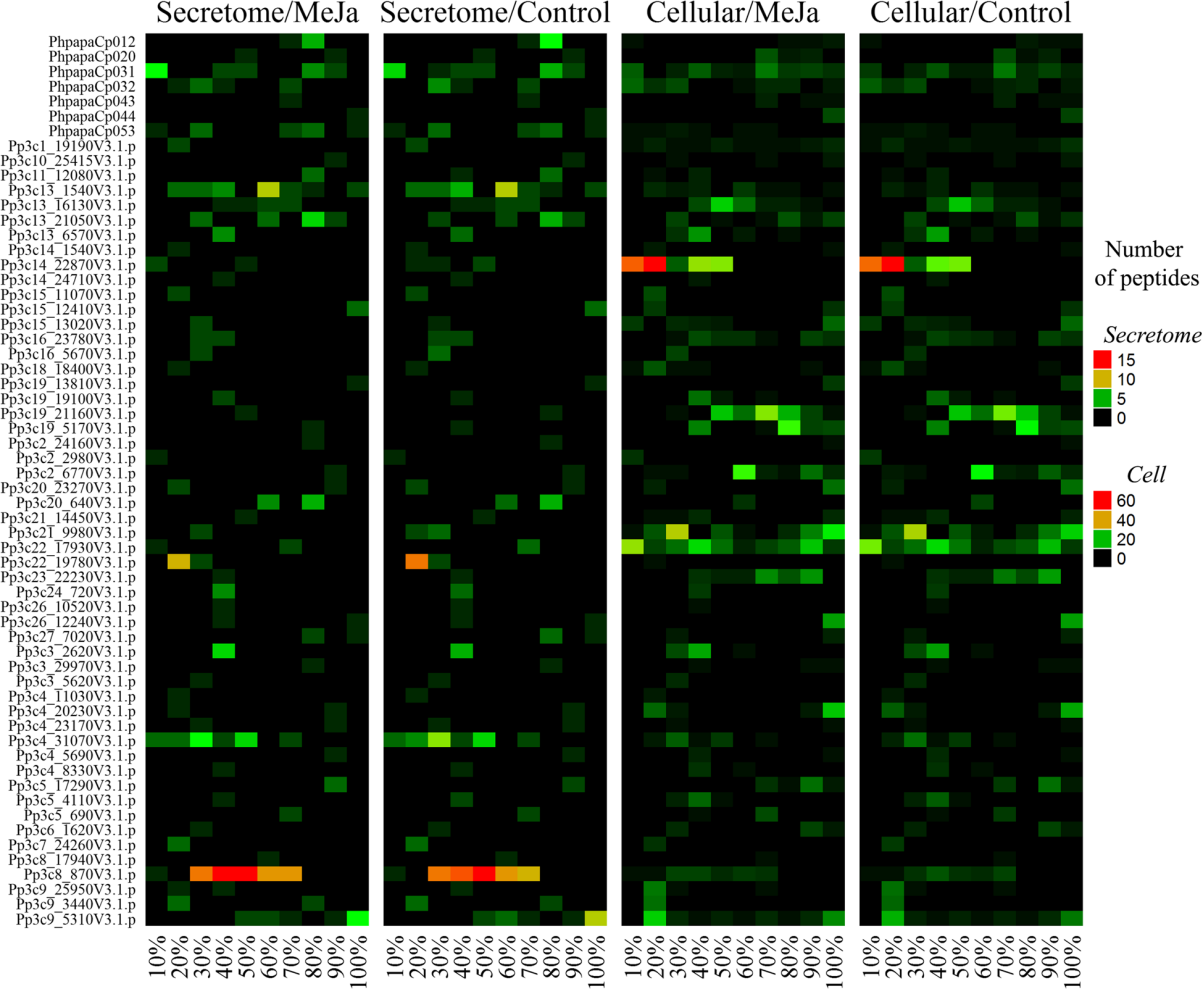
Heatmap showing protein degradation patterns for proteins found in the secretome and cellular peptidome data. Colors correspond to the number of endogenous peptides. X-axis shows the percentage corresponding to portions of the entire protein

We compared the protein degradation patterns (PDPs) of precursors identified in both control and MeJA-treated samples. Taking into account the possibility of generating endogenous peptides from the same parts of proteins that slightly differ from each other (peptide ladders), we used a 10% window for precursor length to estimate PDPs. We found that PDPs were quite similar between MeJA-treated and control samples for the secretome as well as the intracellular peptidome (Fig. 2). We estimated that the fraction of peptides originating from precursors unique to MeJA-treated samples represents less than half of all unique peptides. In other words, hormone-induced stress increased the proteolysis rates of proteins from which peptides are normally formed in the moss peptidome, as well as new protein precursors (Additional file 4: Table S2, Additional file 7: Table S3). Whether this process results from a general disturbance of proteolytic activity or whether the peptides are generated as a target response to stress is currently unknown.

**Fig. 3:**
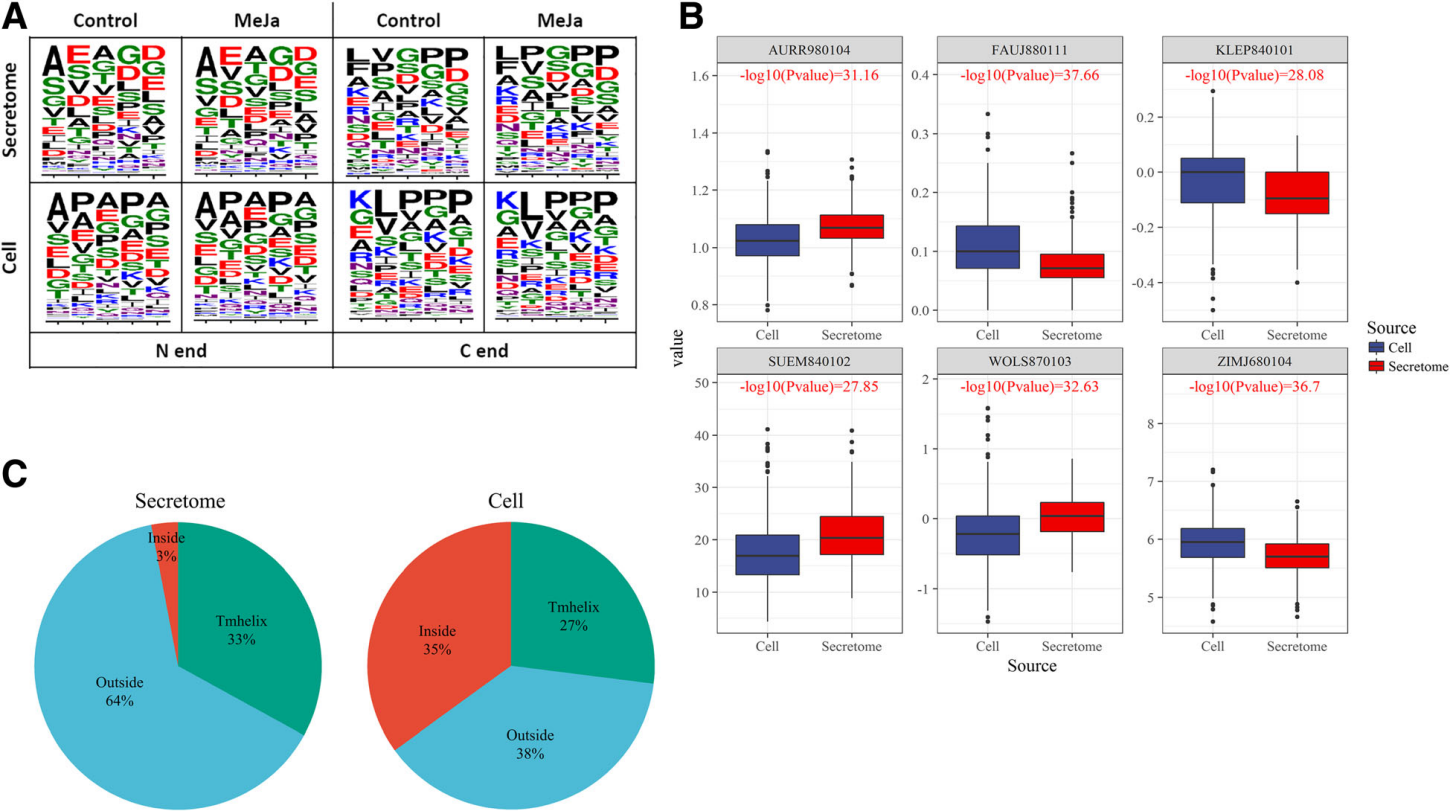
Physicochemical properties of the intracellular and secreted peptides. **a** Sequence logos of the five N- and C-terminal amino acids of peptides from the secretome and cell. Colors correspond to amino acid chemical properties (green, purple, blue, red and black correspond to polar, neutral, basic, acidic and hydrophobic properties, respectively). **b** Boxplots showing differences in mean values of six the most significant “sigAA” indexes (*P*-value differences < e-25). KLEP840101: Net charge [83], FAUJ880111: Positive charge [84], ZIMJ680104: Isoelectric point [85], WOLS870103: Principal property value z3 [86], AURR980104: Normalized positional residue frequency at helix termini N’ [87] and SUEM840102: Zimm-Bragg parameter sigma × 1.0E4 [88]. **c** Locations of peptides identified in the secretome and cell peptidomes for the respective precursor proteins with membrane-spanning alpha helices (TMhelix)

Analyzing shifts in the amino acid compositions of peptides can shed light on their biochemical properties and possible functions. We therefore compared amino acid frequencies (AAFs) in the secretome, cellular peptidome and a set of peptides artificially generated from protein regions not present in the mass-spectrometry data. No significant differences were found between the MeJA-treated and control peptidome in both the secretome and cell, which is well correlated with the similarity in protein degradation patterns. Since the overall amino acid frequency value does not reflect the order of amino acids in a sequence, we then compared the compositions of five-amino-acid regions in the N- and C-termini of the identified peptides (Fig. 3a). MeJA treatment did not significantly alter the amino acid patterns in the C- or N-termini. However, the terminal amino acid patterns differed significantly between the secretome and cellular peptidomes (Fig. 3a). These differences might result from the distinct types of proteolytic enzymes located in these compartments. MeJA treatment only slightly altered PDPs while increasing the proteolysis rate and stimulating the degradation of new protein precursors.

### Physicochemical properties of the intracellular and extracellular peptidomes differ

We investigated whether the physicochemical properties of the extracellular and intracellular peptides differ. Using the AAindex database, 544 indexes were calculated for each amino acid in a peptide, and the mean value was calculated [41]. A comparison of intracellular and extracellular peptides revealed significant differences (“Mann-Whitney” test, *P*-value < e-5) for 197 (36%) indexes. Moreover, 56 AAindexes differed between the secretome and cellular peptidome with *P*-value < e-15 (“sigAA” indexes). Among six “sigAA” indexes (Fig. 3b) showing the most pronounced differences between cell and secretome peptides (*P*-value < e-25), three indexes (KLEP840101, ZIMJ680104 and FAUJ880111) indicated that the secretome peptides tend to have less positively charged amino acids then cell peptides. We then performed Ward’s hierarchical clustering of all 544 indexes and identified six clusters. A cluster comprising AAindexes reflecting the hydrophobicity of amino acids was enriched in the secretome (Fisher’s exact test, *p*-value < 0.05). For example, secretome peptides had significantly higher partition coefficients [42], hydrophobicity [43], transfer energy and organic solvent/water coefficients [44], as well as lower partition energy [45], which are well-known hydrophobicity indexes [46], compared to cellular peptides.

To gain further insight into differences in precursor proteins for the peptides found in the cells vs. the secretome, we predicted membrane-spanning alpha helices (TMhelix) and their orientations in the precursor proteins for these peptides. Similar portions of protein precursors were predicted to contain membrane-spanning alpha helices in the cell and secretome (27% and 33%, respectively) (Fig. 3c). However, peptides originating from the regions of proteins predicted to be located inside the cell were significantly overrepresented in the cell peptidome (Fisher’s exact test, *P*-value = 1.047e-07). In contrast, peptides originating from the regions of proteins predicted to be located outside the cell membrane were significantly overrepresented in the secretome (Fisher’s exact test, *P*-value = 0.006516). These results provide additional evidence that the identified peptide pool is indeed a part of the secretome rather than an artifact related to sample preparation.

### Methyl jasmonate induces antimicrobial activity of the moss secretome

**Fig. 4:**
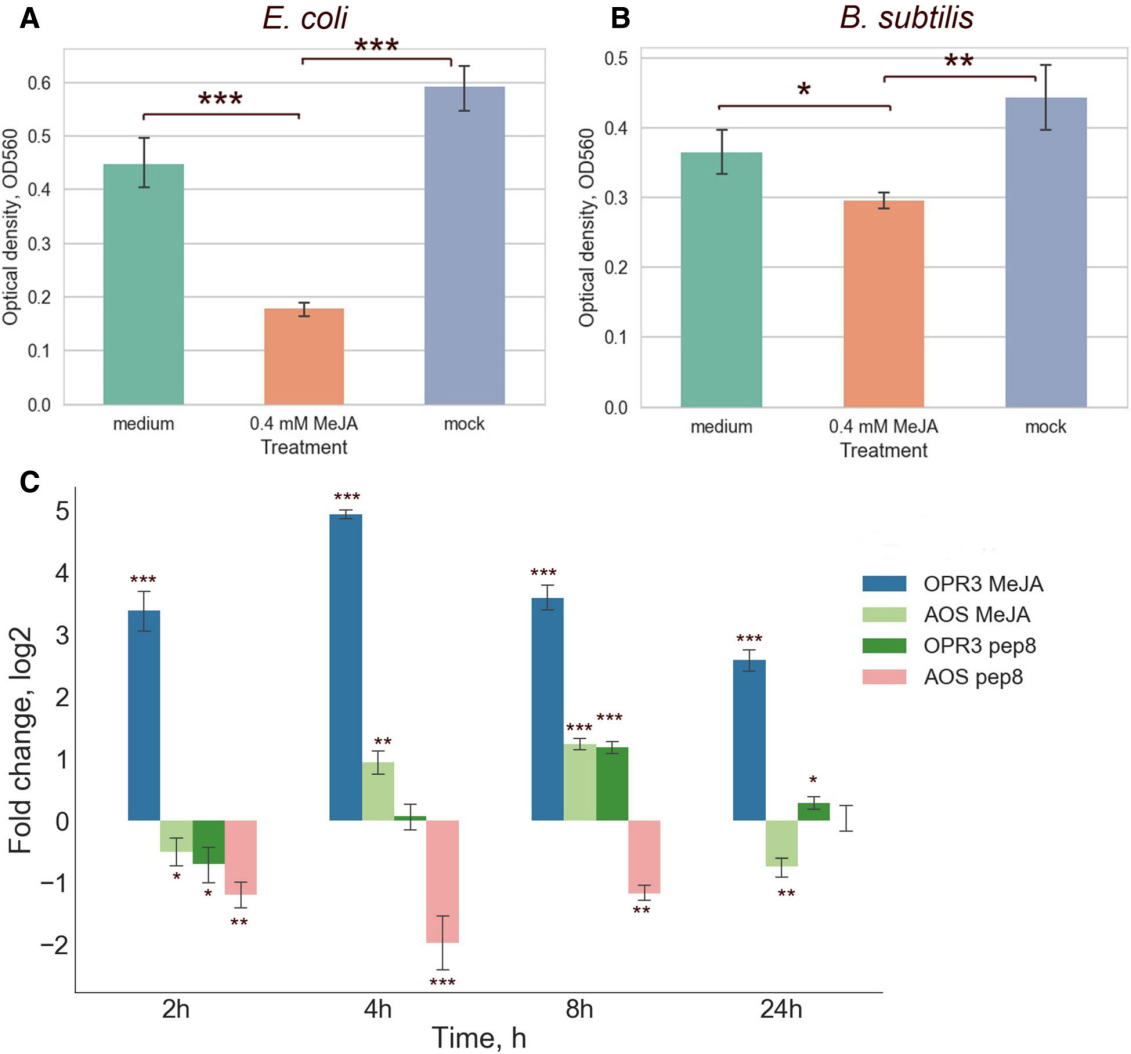
Analysis of biological activity of the secreted peptides. **a** The barplot shows optical density of *E. coli* cultures after 24-h incubation with the 400 μM MeJA-treated, mock secretome samples and culture medium with 400 μM MeJA. **b** The barplot shows optical density of *B. subtilis* cultures after 24-h incubation with 400 μM MeJA-treated, mock moss secretome samples and culture medium with 400 μM MeJA. The bars (M ± SD) represent the results of three independent experiments performed in triplicate. **c** qRT-PCR analyses of OPR3 and AOS genes expression in protonema tissue treated with 1μM pep8 and 0.4 mM MeJA. *P* value were calculated by an unpaired t-test. ^*^P<0.05; ^**^P<0.005; ^***^P<0.0005

Peptides play an important role in immune responses in plants. However, little is known about cryptic peptides, which have antimicrobial activity and are generated as a result of the degradation of functionally active proteins. Since the secretome is the first frontier in plant pathogen interactions, we directly tested antimicrobial activity of MeJA-treated secretomes using a serial dilution method with *E. coli* and *B. subtilis* bacteria (see Methods for details). The one-hour MeJA-treated secretomes restricted the bacterial growth (bacteriostatic effect) in comparison with untreated samples and cultural medium with addition of 0.4 mM MeJA (Fig. 4a and b), suggesting a possible role of endogenous peptides as a quick-released antimicrobial agent. Some oxylipins have antimicrobial activity, we therefore additionally tested antimicrobial activity of secretomes treated with different concentration of MeJA (0.05 mM, 0.4 mM and 1 mM; Additional file 8: Figure S5). The most pronounced bacteriostatic effect was observed with 0.05 and 0.4 mM MeJA-treated secretome, implying a regulatory role of MeJA rather than functioning as an antimicrobial compound.

We than performed additional experiments to determine, if these results might reflect a release of antimicrobial endogenous peptides during protein degradation. For this, the protease inhibitor cocktail, preventing proteolytic degradation of secreted proteins in tissue culture media, was applied during 1-h MeJA treatment. A significant decrease of the bacteriostatic effect was observed (an unpaired t-test, *P*< 0.0005; Additional file 9: Figure S6), suggesting a possible role of endogenous peptides as a quick-released antimicrobial agent.

We then estimated the potential antimicrobial activity of peptides identified in the cell (Additional file 10: Tables S4) and secretome (Additional file 11: Table S5) using three bioinformatic tools: *i*AMPpred, CAMP and ADAM (see Materials and Methods). We predicted that 3.5% of all peptides from the secretome might have antimicrobial properties based on data from all three servers. These peptides originated from various groups of protein precursors, including Pp3c2_24160 (glyceraldehyde-3-phosphate dehydrogenase), Pp3c14_17710 (cucumicin) and others. In the culture medium, the most overrepresented peptides were fragments of α-expansin protein. Some of these peptides have potential antimicrobial activity, such as the peptides IHNVGGAGDVVSVK and TDINLDLGDGKQG. Treatment with MeJA led to the appearance of potential AMPs originating from some extracellular proteins, such as Pp3c4_16840 (FASCICLIN-LIKE ARABINOGALACTAN PROTEIN 4) and Pp3c25_3840 (PECTINESTERASE 8-RELATED). We also found that the abundance of the peptide with the highest antimicrobial potential, INIINAPLQGFKIA (pep8, FC_log2_ = 1.3), increased upon MeJA-treatment.

We also analyzed peptide pools identified in the cell peptidome and predicted 4% (173) as potential antimicrobial peptides. We found an increase in the number of potential antimicrobial peptides (AMPs) in tissues treated with MeJA vs. the control (+10 peptides). We also found that the abundance of 37 (21 %) peptides with predicted antimicrobial activity in protonemata was up-regulated (log_2_FC >1) after MeJA-treatment.

### Analysis of the biological activity of synthetic peptides

To verify the biological activity of the predicted AMPs, we selected eight peptides (five from cell peptidome and three from secretome) based on the following criteria: physicochemical properties, high predicted antimicrobial potential and changes in abundance upon MeJA treatment compared with the control (Table 1). We investigated the antimicrobial activity of the selected peptides *in vitro* using a serial dilution method with *E. coli* and *B. subtilis* bacteria. We found that the minimum inhibitory concentration (MIC) for two peptides, LVQIGTKIVGVGRNYAAH (pep1, a fragment of fumarylacetoacetate hydrolase domain-containing protein) from the cell and INIINAPLQGFKIA (pep8, a fragment of the predicted protein) from the cell secretome were 64 and 16 μg/mL, respectively (Table 2, Additional file 12: Figure S7). As a positive control, a well-known antimicrobial peptide melittin was used [47]. We obtained a similar MIC for pep8 and melittin (16 and 8 mkg/ml, respectively), suggesting a possible role identified peptide as an antimicrobial agent. In addition, the cellular peptide KIKVAINGFGRIG (a fragment of the glyceraldehyde-3-phosphate dehydrogenase (GAPDH)), inhibited bacterial growth at a concentration of 128 μg/mL on day 1 after inoculation.

**Table 1:** List of peptides used for synthesis and verification of antimicrobial activity

№	Peptide sequence	Protein	Gene ID	*i*AMPpred index Antibacterial	Fold change, log2
Intracellular					
1	LVQIGTKIVGVGRNYAAH	Fumarylacetoacetate hydrolase domain-containing protein 1	Pp3c9_26130V3	0.832	4.2
2	AAQGQKIENTKLAGAAGDILSGLAAYGKLD	Predicted	Pp3c22_17930V3	0.884	0.9
3	VAAVAPKFATLKPLG	Chloroplast chaperonin 21	Pp3c19_4270V3	0.811	0.7
4	KIKVAINGFGRIG	Glyceraldehyde-3-phosphate dehydrogenase	Pp3c2_24160V3	0.998	-1.9
5	IVPTSTGAAKAVALVLPNLK	Glyceraldehyde-3-phosphate dehydrogenase	Pp3c2_24160V3	0.597	3.1
Secretome					
6	INIINAPLQGFKIA	Predicted	Pp3c14_22870V3	0.941	1.3 (secretome) 4.1 (cell)
7	TDINLDLGDGKQG	alpha expansin protein family EXPA6	Pp3c8_870V3	0.513	1.6
8	VVDLLAPYRRGGKIG	Predicted	PhpapaCp032	0.507	-3.2

**Table 2:** Minimum inhibitory concentration (MIC) values of peptides

Peptide sequence	*E. coli*		*B. subtilis*	
	MIC, μg/mL	% inhibition	MIC, μg/mL	% inhibition
LVQIGTKIVGVGRNYAAH	64	>90%	64	>90%
INIINAPLQGFKIA	16	>90%	32	>90%

Plant peptides can play a role as damage- or danger-associated molecular patterns (DAMPs) and activate immune responses [48, 49]. We used quantitative RT-PCR analysis (qRT-PCR) to check the ability of synthetic peptides with antimicrobial activity to induce the transcription of known pathogenesis-related genes, such as allene oxide synthase (AOS), OPDA reductase (OPR3) and phenylalanine ammonia lyase (PAL). According to the previous articles, the transcription of OPR3 is induced by MeJA and pathogens, and expression of PAL is enhanced both by salicylic acid (SA) and MeJA [38–40]. In our study, we observed a clear response of OPR3 to treatment with MeJA, but not SA (Additional file 13: Figure S8). Out of the peptides with antimicrobial activity and all secretome peptides, qRT-PCR showed that the peptide INIINAPLQGFKIA regulates transcription of the OPR3 and AOS genes (Fig. 4c). In parallel, moss protonema was treated with 0.4 mM MeJA as a positive control. We compared the effect of exogenous MeJA on the expression of the OPR3 and AOS genes and found a similar pattern, suggesting additional regulatory role for the peptide (Fig. 4c).

Therefore, our results indicate that some components of the peptide pools have clear antimicrobial and signaling activity. Moreover, treatment with stress hormones can increase the number of potential AMPs and the abundance of such peptides in the cell and secretome.

## Discussion

Protein degradation in plants leads to the generation of thousands of different peptides. While some of these peptides are simply intermediate products in protein degradation, others have distinct biological functions inside or outside the cell [7, 11–14]. In this study, we analyzed the secretome and cellular peptidomes of the moss *P. patens* under normal conditions and upon treatment with the stress hormone – methyl jasmonate. The global protein degradation patterns of common proteins were significantly different between the cell and the secretome, suggesting a possible difference in the sets of proteolytic activity located in these compartments. Moreover, some of the secreted and intracellular peptides showed clear biological activity. Our data point to the relevance of “active management” of peptide pools in plants, and suggest that the products of protein breakdown are not just passive but contain important biologically active elements. Our research highlights the need for further studies of peptide pools and the biological functions of these peptides in plants.

### Stress hormones influence protein degradation pathways

Plants contain two major degradation pathways that carry out digestive proteolysis: autophagy and the ubiquitin–proteasome system (UPS) [50, 51]. Some studies have shown that oxygenic stress can inhibit 26S proteasome activity, thereby increasing the activity of the 20S proteasome, which degrades oxygenated proteins [52]. Moreover, UPS is targeted by pathogens to enhance virulence [53]. For example, *Pseudomonas syringae* pv. *tomato* (Pst) secretes effector proteins to suppress proteasome activity and block SA signaling [53, 54]. In addition to the UPS, stress conditions or pathogen attack activate various proteases in the apoplast, including phytaspase [55, 56]. Our results show that stress hormone treatment leads to an increase in the number of unique peptides in the moss protonemata. These peptides are released as a result of degradation of new proteins (phytohormone-induced proteolysis) and changes in the degradation patterns of existing precursors. The latter process leads to the generation of peptide ladders of non-identical but similar peptides between control and MeJA-treated cells.

Thus, stress hormones do not merely induce the degradation of specific repressors of transcription factors, but they also alter the peptide pools in the cell and secretome. Responses to biotic stress appear to include the phytohormone-driven proteolysis of functional proteins, which can lead to the formation of bioactive peptides, e.g., those with antimicrobial activity.

### Moss secretome comprises hundreds of different peptides

Using mass-spectrometry analysis, we identified approximately 500 endogenous peptides generated from functional proteins in liquid culture medium during protonemata growth. We found that MeJA treatment altered the peptide profile of the secretome. The corresponding protein precursors are involved in processes including responses to oxidative stress, polyamine metabolism, cell wall biogenesis and so on. MeJA treatment led to the induction and secretion of peptides from various groups of stress proteins. For example, upon MeJA-treatment, fragments from protein precursor PR10 (Pathogenesis Related protein), 70 kDa heat shock protein and MYC transcription factor were detected in the secretome, whereas these three peptides were absent in control samples. Earlier, it has been shown, that PR1 protein from tomato contains cryptic bioactive peptide – CAPE1 [14]. This finding supports the notion that MeJA can induce the biosynthesis of biologically active peptides in plant cells, which plays an important role in stress responses.

A previous proteomic analysis of the *P. patens* secretome showed that treatment with chitosan induced the secretion of proteins such as α-expansin, pectin methylesterase, photosystem II, lipoxygenase and LEA proteins [37]. We detected peptides from these proteins in both control and MeJA-treated cells. Other study has recently reported the proteome composition of *P. patens* bioreactor supernatants [36]. A complex extracellular proteolytic network including different types of proteases as well as their inhibitors were identified in moss secretome. The contribution of these proteases to the process of extracellular peptidome formation is subject for further evaluation. A slight overlap (Additional file 14: Figure S9) between precursors of the extracellular peptides from our study and secretome proteins from Hoernstein et. al. dataset suggests our conclusions that the extracellular peptidome is not merely a byproduct of extracellular proteins degradation.

Interestingly, we detected only a slight overlap between the secretome and cell peptidome, which raises the question of why such differences occur. First, because protein precursors in the secretome include extracellular and membrane proteins, one of the sources of secretome-specific peptides might be specific extracellular degradation pathways [57, 58]. Second, some peptides with an intracellular origin could be generated from intracellular proteins inside the cell and then exported into the secretome [57], while their concentration in the cell would be negligible. Third, while some proteases are common to the secretome and cell, the proteins subjected to their action might differ between the cell and secretome.

### Mechanisms of peptidome generation might be conservative

Recent studies on mammals showed that the cell peptidome comprises a large number of intracellular peptides originated from functional proteins and probably produced by the UPS [59, 60]. In a eukaryotic cell, the majority of 3-25 aa intracellular peptides are formed by the proteasome [59, 61–66]. Most of the peptides identified in the current study are approximately 15 amino acids in size, indicating that they are possible products of the ubiquitin–proteasome system (UPS) [50, 51]. Interestingly, proteasome inhibitors can alter the pool of endogenous peptides, pointing to a dynamic nature of cell peptidome formation [67].

Some principles of intracellular peptide formation, such as over-representation of N- and C-terminal fragments, lack of correlation between protein levels and protein degradation fragments suggest a complex picture of cell peptidome formation [68]. For example, about 40% of intracellular peptides from mouse brain belong to N- (21%) or C-terminal (21%) regions of proteins [60, 68]. According to our results, about 40 % of total peptides in moss cells were raised from C-terminal fragments of proteins while that was not true for secreted peptides. Peptides from mitochondrial proteins constitute approximately 1/4 of the identified intracellular peptides in mouse brain [60, 68]. In our study, most intracellular peptides originated from proteins are involved in photosynthesis, the Calvin cycle, glycolysis and sucrose biosynthesis (Fig. 1b). It seems that organelles, such as mitochondria in mammalian cells and chloroplasts and mitochondria in plants are sources of a large number of intracellular peptides. These findings suggest a great similarity in principles of peptide pools formation in plant and animal cells.

It is interesting to note that intracellular mammalian peptides are extensively secreted, implying their possible functions in cell-cell communication [60, 69]. We revealed that about 40% of secreted peptides in moss constitute intracellular peptides or their shorter versions. Despite this, amino acid compositions and physicochemical properties significantly differ between the cell and the secretome peptidomes in moss.

Besides peptides from functional proteins, secretome peptidome in plant and animal encompass a number of post-translationally modified peptide hormones, which regulate different aspects of response to environmental stimuli [20, 69]. Taken together, these findings suggest that secretome peptidome of animal and plant cell is a complex mixture of specialised peptide hormones and fragments of functional proteins, including full-size or shorter intracellular peptides. Such common features of mammalian and plant peptidomes might indicate a certain conservative mechanism of petidome formation, the whole nature of which is yet to be revealed.

### Functional proteins are source of bioactive peptides

Plant secretome is a source of bioactive peptides, which are induced by stress hormones. In addition to their regulatory functions, secreted peptides can also have antibacterial, antifungal and insecticidal functions [22]. A number of studies have shown that functional proteins can be source of antimicrobial peptides [70–72]. Our study shows that MeJA treatment led to an increase in the overall number of endogenous peptides, including peptides with predicted antimicrobial activity. We showed that the peptide KIKVAINGFGRIG, a fragment of glyceraldehyde-3-phosphate dehydrogenase (GAPDH), inhibited bacterial growth. GAPDH-derived antimicrobial peptides have been also found in *Saccharomyces cerevisiae* and skipjack tuna [73, 74]. These findings suggest a possible role of endogenous peptide pool, released from functional proteins, as a source of bioactive molecules, including antimicrobial ones.

In recent studies, a range of peptides regulating plant immune response has been identified [12, 48]. Interestingly, we found that moss secreted peptides might have antimicrobial and regulatory activities. We identified the peptide INIINAPLQGFKIA, which has the highest predicted antimicrobial potential in the secretome, induced transcription of OPR3 gene 8h after treatment and inhibited the transcription of AOS gene in a time-dependent manner. The level of transcription of OPR3 increases under pathogen attack, suggesting a possible role of this peptide in immune response [39]. Previous studies of immune signaling peptides have also shown a time-dependent manner of defense gene transcription upon synthetic peptide treatment [12, 14]. Therefore, our observations lead to the hypothesis that endogenous peptides could represent a rapidly available resource of bioactive peptides with which the plant responds to stress.

## Conclusion

We have presented a comprehensive analysis of the endogenous peptides in both the cells and the secretome of the moss *P. patens* before and after treatment with methyl jasmonate. Peptide pools in the cells and secretome significantly differ in amino acid composition and physicochemical properties. Our data point to a significant alteration of peptide pools in both the secretome and the cells after hormone treatment. This alteration correlates with hormone-specific proteolysis of new functional proteins, along with minor changes in the degradation patterns of existing precursors. We also detected antimicrobial activity in a number of the peptides generated from functionally active proteins upon hormone treatment. Therefore, our data point to the relevance of “active management” of peptide pools in plants, and suggest that the products of protein breakdown are not just passive but contain important biologically active elements. Our research highlights the need for further studies of peptide pools and the biological functions of these peptides in plants.

## Methods

### Plant materials and treatments

Protonemata of the moss *P. patens* subsp. *patens* (“Gransden 2004”, Freiburg) were grown in 200 mL liquid Knop medium with 500 mg/L ammonium tartrate under white light with a photon flux of 61 μmol/m^2^•s under a 16-h photoperiod at 24°C. For mass spectrometry analysis, five-d-old protonema tissue was treated with 0.4 mM MeJA and incubated for 1 h. To verify the effect of exogenous MeJA on the expression of pathogenesis-related genes, protonema tissue was incubated with 0.05, 0.1, 0.4 and 1mM MeJA or 0.4 mM salicylic acid (SA) for 1 h under white light. To analyze antimicrobial activity of secretome five-d-old- protonema tissue was treated with different concentration of MeJA. Treatment with cocktail of protease inhibitors was performed according to the dilution 1:200, as recommended by the manufacturer (P1860 Sigma Aldrich, USA). At least three independent biological replicates were used for each type of analysis. To test the gene expression induced by the peptides, five-d-old protonema tissue was treated with either mock solution or 1 μM peptide solution and harvested after 2, 4, 8 and 24 h later. The experiments were preformed in four independent biological replicates with three technical replicates.

### Peptide extraction

Intracellular peptides were extracted from moss protonemal tissue as previously described with minor modifications [7]. For peptide extraction from moss tissues, the extraction solution contained 1 М acetic acid in 10% acetonitrile and 10 μL/mL protease inhibitor cocktail (Sigma-Aldrich, USA). The ground material was placed into cooled extraction solution containing proteinase inhibitor cocktail (Sigma-Aldrich, USA; inhibits serine, cysteine, and aspartic proteases, metalloproteases and aminopeptidases) and homogenized using a Dismembrator S ball mill (Sartorius, Göttingen, Germany) at 2,600 rpm for 2 min with a mixture of glass balls 0.1, 0.3 and 1 mm in diameter (Sartorius). The suspension was centrifuged at 11,000 × *g* for 10 min at 4°C. The supernatant was transferred to a clean test tube and centrifuged again at 11,000 × *g* for 10 min at 4°C, after which the pellet was discarded. The samples were immediately placed onto a gel filtration column to extract and fractionate the peptides. Gel filtration was carried out on a ХK 26/40 GE Healthcare Life Science column filled with sorbent Sephadex G25. Elution was performed with 25 mM Tris-HCl, 0.15 mM NaCl at a flow rate of 1.5 mL/min. Proteins and peptides were detected on an AKTA pure 25 (GE Healthcare) device at a wavelength of 280 nm. The fraction containing peptides was lyophilized and resuspended in 100 μL 5% acetonitrile-0.1% trifluoroacetic acid, followed by desalting in microcolumns with two С18 disks (Empore).

Extracellular peptides were extracted from 400 mL protonemata culture medium (Knop medium with 500 mg/L ammonium tartrate). The culture medium was filtered through a 0.22 μm membrane filter (Millipore), lyophilized and resuspended in 500 μL 5% acetonitrile - 0.1% trifluoroacetic acid, followed by centrifugation at 10,000 × *g* for 10 min. Peptides were isolated from the culture medium by solid-phase extraction on reverse-phase DSC18 cartridges (Discovery DSC-18, Supelco, USA) using 500 μL 50% and 500 μL 80% acetonitrile solutions for the elution. The eluted peptides were concentrated in a SpeedVac and resuspended in 15 μL 5% acetonitrile-0.1% trifluoroacetic acid. The pool of peptides was isolated by solid-phase extraction in ZipTip Pipette tips (Millipore) using a 30 μL 50% acetonitrile solution for the elution. Eluted peptides were concentrated in a SpeedVac and resuspended in 15 μL 5% acetonitrile-0.1% trifluoroacetic acid, followed by mass-spectrometry analysis of all prepared samples.

### Total RNA Isolation and qRT-PCR

Total RNA from protonemata was isolated as previously described [75]. Quality and quantity were evaluated using electrophoresis on agarose gel with ethidium bromide staining. Total RNA concentration of samples was precisely measured using the Quant-iT™ RNA Assay Kit, 5–100 ng on a Qubit 3.0 (Invitrogen, US) fluorometer. cDNA was synthesized using the MMLV RT kit (Evrogen, Russia) according to the manufacturer’s recommendations. Random hexamer primers were used to prepare cDNA from 2 μg total RNA after DNase treatment. Primers were designed using the PrimerQuest Tool (http://eu.idtdna.com/Primerquest/Home/Index) (Additional file 15: Table S6). Real-time PCR was performed using the BioMaster HS-qPCR (2×) system fluorescent probes (Biolabmix, Russia) on a LightCycler® 96 (Roche, Mannheim, Germany). qRT-PCR was carried out in three biological and three technical replicates. cDNA representation was normalized using stably transcribed reference gene actin 5 (Pp1s381_21V6.1). The normalized ratios were obtained using the LightCycler® 96 software. Control samples were used as a calibrator.

### LC-MS/MS analysis and peptide identification

Mass-spectrometry analysis was performed in at least three independent biological and three technical repeats. Reverse-phase chromatography was performed with an Ultimate 3000 Nano LC System (Thermo Fisher Scientific), which was coupled to a Q Exactive HF mass spectrometer (Q Exactive^TM^ HF Hybrid Quadrupole-Orbitrap^TM^ Mass spectrometer, Thermo Fisher Scientific, USA). Peptide samples were loaded on a Chrom XP C18 trap column (3 μm, 120 Å, 350 m×0.5 mm ; Eksigent, Dublin, CA) at a flow rate of 3 ml/min for 10 min and eluted through a 15-cm long C18 column with a diameter of 75 μm (3μm, Acclaim® PepMap™ RSLC, Thermo Fisher Scientific, USA). The injection volume was 5 μL and the temperature of the autosampler was kept at 4°C. The peptides were loaded in mobile phase A (0.1% (v/v) formic acid) and eluted with a linear 5–35% gradient of mobile phase B (80% acetonitrile, 0.1% formic acid) at a flow rate of 0.3 μL/min. Total run time included initial 10 min of column equilibration to mobile phase A, then a linear 5–35% gradient of mobile phase B over 65 min, 5 min to reach 99% mobile phase B, flushing 5 min with 99% mobile phase B and 5 min re-equilibration with mobile phase A. Column temperature was kept at 40°C. Mass spectra were acquired at a resolution of 60,000 (MS) and 15,000 (MS/MS) in a range of 400–1,500 m/z (MS) and 200–2,000 m/z (MS/MS). An isolation threshold of 67,000 was determined for precursor selection and (up to) the top-10 precursors were subjected to fragmentation with high-energy collisional dissociation (HCD) at 25 NCE and 100 ms activation time. Other settings: charge exclusion - unassigned, 1, >6; peptide match – preferred; exclude isotopes – on; dynamic exclusion - 20 s was enabled.

Mass-spectrometry data were searched with MaxQuant v1.5.8.3 against a database containing the protein sequences from Phytozome v12.0 merged with chloroplast and mitochondrial proteins (33053 entries) and sequences from a database of common contaminant proteins [76]. The MaxQuant protein FDR filter was disabled, while the peptide FDR remained at 1%. The parameter “Digestion Mode” was set to “unspecific”, and modifications were not allowed. All other parameters were left at default values. Features of PSMs (length, intensity, number of spectra, Andromeda score, intensity coverage and peak coverage) were extracted from MaxQuant’s msms.txt files. Statistical analysis including the quantification of peptides based on extracted ion chromatograms (XICs) values was performed in Perseus (v1.6.0.7) [77].

### Retention Time (RT) peptides identification by MRM-MS

For MRM, 6 pmol of retention time (iRT) peptides mix (Biognosys, Switzerland) was added to 200 mL of a liquid medium or to 5 mL of extraction solution before intracellular peptides isolation. MRM-MS analysis was performed on on QTRAP 4500 (Sciex, USA) triple quadrupole mass spectrometer equipped with a NanoSpray III ion source (Sciex, USA) coupled to a nanoLC Ultra 2D+ nano-HPLC system (Eksigent, USA). The HPLC system was configured in a trap-elute mode. A mixture of 98.9% water, 1% methanol, and 0.1% formic acid (v/v) was used as the sample loading solution and mobile phase A. Mobile phase B was 99.9% ACN, 0.1% formic acid (v/v). Samples were loaded on a trap column (10 mm, 100 um i.d., Aeris Peptide XB-C18, 2.6 uM, 100 A) at a flow rate of 2.5 μL/min over 10 min and eluted through the separation column (20 cm, 75 um i.d., Aeris Peptide XB-C18, 2.6 uM, 100 A) at a flow rate of 300 nL/min. Total run time including initial 10 min of column equilibration to mobile phase A (0.1% formic acid), then gradient from 10–40% mobile phase B over 30 min, 2 min to reach 95% mobile phase B, flushing 10 min with 95% mobile B and 5 min re-equilibration to mobile phase A 15 min. The chromatographic columns were thermostated at 50 °C.

Target peptides were measured in duplicate, which were averaged and total fragment peak area and normalized to each peptides’ mean signal was used as a proxy of peptide abundances. Transition list was created after evaluation of all theoretical b and y fragments of charge +1 of precursor ions of charges +2 and +3. Top 5 transitions of most intense precursor ions were used for further measurements after analysis of synthetic peptide samples. MRM data was acquired in positive ion mode in non-scheduled MRM method with 1.49 s total scan time and dwell time 10 msec (Q1 and Q3 resolution set to “UNIT” - 0.7 Da FWHH), pause between mass ranges was 5 ms). Processing of the data included peak selection (with manual review for interference and missing signals), peak integration and export utilizing Skyline software. Further analysis was performed by a homemade script in R.

### Prediction of antimicrobial activity

The antimicrobial potential of peptides was predicted based on sequence using three servers: CAMP (http://www.camp3.bicnirrh.res.in), *i*AMPpred (http://cabgrid.res.in:8080/amppred/) and ADAM (http://bioinformatics.cs.ntou.edu.tw/ADAM/links.html), which use machine-learning algorithms (support vector machines [SVM]) to predict the antimicrobial activity in a peptide. For CAMP and *i*AMPpred, a threshold value of 0.5 was used to predict antimicrobial activity. For ADAM, a threshold value of 0 was used to predict antimicrobial activity. Only antimicrobial peptides with a probability of misclassification less than 5% were used.

### GO enrichment analysis

The topGO bioconductor R package was used to estimate GO enrichment (Alexa and Rahnenfuhrer, 2010) using the Fisher’s exact test in conjunction with the “classic” algorithm (false discovery rate [FDR] < 0.05). Gene Ontology (GO) terms assigned to *P. patens* genes were downloaded from Phytozome. Only GO terms containing > 5 genes in a background dataset were considered in the enrichment analysis. Redundant GO terms were removed using the web-based tool REVIGO (Supek et al., 2011).

### Statistical analysis and visualization

All statistical calculations were performed using R programming language. For continuous variables without normal distribution, the nonparametric “Mann–Whitney–Wilcoxon U-Test” test was used while Student’s t-Test was applied to normally distributed data. To visualize the results histograms and box plots were built by ggplot2 library in R [78], the Venn diagrams were drawn using the VennDiagram library in R [79]. PDP heatmaps were drawn by ComplexHeatmap library in R, using the matrix prepared by a custom python script - Degradome.py. Matrix for PDP analysis were constructed by splitting a protein sequence into 10 equal windows followed by counting the number of overlaps between the window coordinates and start coordinates of MS peptides corresponding to the protein. To estimate amino acid biases custom python script (AAbiases.py) was applied. The calculation of physico-chemical properties of peptides was accompanied by AAIndex.py script (http://pydoc.net/pydpi/1.0/pydpi.protein.AAIndex/). Logos pictures were generated by WebLogo service [80]. All python scripts used in this work are available at GitHub repository (https://github.com/Kirovez/Secretome).

### Peptide synthesis

The peptides were synthesized by Shanghai Ruifu Chemical Co., Ltd. (Shanghai, China). The purity of the peptides was >95%, and their molecular weights were confirmed by mass-spectrometry analysis. The peptides were resuspended in sterile water to a concentration of 2,560 μg/mL and stored at -80°C.

### Antimicrobial activity assays

Antimicrobial activity was determined in 96-well polypropylene microtiter plates (Corning, cat. no. CLS3879, Corning, USA) using the standard microtiter dilution method in accordance with general practice [81]. The two bacterial strains — *Escherichia coli* (K-12 substr. MG1655) and *Bacillus subtilis* (168HT) — were stored with 10% glycerol at -70 °C until use. Bacterial cultures were grown in LB medium for 16 h at 37°С, and 100-μL aliquots of culture were used to inoculate 9.9 mL Mueller-Hinton Broth (MHB, Difco; Becton Dickinson Diagnostics) medium (Beef extract 2g/L, Acid digest of Casein 17.5g/L, Starch 1.5g/L). The cultures were incubated on a shaker to a final concentration of 1×10^8^ CFU/mL, followed by serial dilution in 2× MHB growth medium to prepare test cultures at a concentration of 1×10^6^ CFU/mL. 50 ml of a lyophilized secretome sample was dialyzed against normal saline (0.9%) for 16 h at 25^0^C using CelluSep H1 membranes (MFPI, USA). Microtiter plate wells each received aliquots of 50 μL of the culture suspension, followed by the addition of 50 μL of the diluted secretome sample. As a control, both pure liquid medium and medium with addition of 400 μM MeJA were used. After incubation at 37 °C for 24 h, the antimicrobial susceptibility was monitored by measuring the optical absorbance at 570 nm using a microplate reader. 1/32 diluted samples were used for visualization of results.

A serial dilution method in sterile 96 Well Collection Plates (Corning 3870) in liquid MHB medium was used to define the minimum inhibitory concentration (MIC) of the peptides against *E. coli* K-12 MG 1655 and *B. subtilis* 168 HT as previously described [81]. Diluted peptide (50 μL) in MHB medium was added to each well at the appropriate concentration (128 to 0.25 μg/mL), along with 50 μL of bacterial suspension at a concentration of 10^6^ CFU/mL of microbial cells. The experiment was carried out in three replicates. Liquid MHB medium without the addition of the peptide used as a negative control. The plates were incubated at 37°С for 20 h without mixing. The MIC of the peptides was assessed visually and by measuring the spectrophotometric absorbance at 570 nm to identify the concentration that inhibited the visible growth of bacteria.

## Additional files

Additional file 1: **Figure S1.** A workflow of the peptidomic analysis. (PDF 257 kb)

Additional file 2: **Figure S2.** The MRM chromatogram of eleven standard RT peptides added in cell and secretome samples. (PDF 613 kb)

Additional file 3: **Table S1.** The list of synthetic RT peptides identified by MRM-MS in cell and secretome samples. (XLSX 11 kb)

Additional file 4: **Table S2.** List of intracellular endogenous peptides identified in control and MeJA-treated protonemata. (XLSX 540 kb)

Additional file 5: **Figure S3.** The distribution of the log2-transormed (log2_FC) peptide intensities based on Xtracted Ion Chromatogram (XIC) values. (PDF 129 kb)

Additional file 6: **Figure S4.** TreeMap showing GO enrichment analysis results for all protein precursors for cell peptides. (PDF 184 kb)

Additional file 7: **Table S3.** List of secreted peptides identified in control and MeJA-treated protenamata. (XLSX 67 kb)

Additional file 8: **Figure S5.** The barplot shows optical density of E. coli and B. subtilis cultures after 24-h incubation with secretome samples treated with different concentration of MeJA. The bars (M ± SD) represent the results of three independent experiments performed in triplicate. (PDF 220 kb)

Additional file 9: **Figure S6.** The barplot shows optical density of E. coli and B. subtilis cultures after 24-h incubation with secretome samples. Secretome+inhibitor+MeJA – secretomes of moss protonema treated with 400 μM MeJA and the protease inhibitor cocktail; secretome+MeJA - secretomes of moss protonemata treated with the 400 μM MeJA; secretome+inhibitor - secretomes of moss protonema treated with the protease inhibitor cocktail. The bars (M ± SD) represent the results of three independent experiments performed in triplicate. (PDF 202 kb)

Additional file 10: **Table S4.** Antimicrobial potential of intracellular peptides predicted using three tools: CAMP, iAMPpred and ADAM. (XLSX 255 kb)

Additional file 11: **Table S5.** Antimicrobial potential of secreted peptides predicted using three tools: CAMP, iAMPpred and ADAM. (XLSX 35 kb)

Additional file 12: **Figure S7.** Analysis of minimal inhibitory concentration (MIC) for the two peptides (Pep1- LVQIGTKIVGVGRNYAAH and Pep8- INIINAPLQGFKIA). Melittin was used as a positive control. The barplot shows optical density of B. subtilis and E. coli cultures after 24-h incubation with different peptide concentrations. (PDF 171 kb)

Additional file 13: **Figure S8.** (A) Results of quantitative polymerase chain reaction (qRT-PCR) for PAL and OPR genes after treatment of protonemata with 50μM, 100μM, 400μM and 1mM methyl jasmonate (MeJA). (B) Results of qRT-PCR for PAL and OPR genes after treatment of protonemata with 10μM, 100μM, 400μM and 1mM salicylic acid (SA); The normalized ratios and standard deviation of three independent triplicate experiments are shown. (PDF 182 kb)

Additional file 14: **Figure S9.** Venn diagram showing a comparison between protein precursors of our control dataset and proteome of P. patens bioreactor supernatants (Hoerstein et. al. 2018). (PDF 250 kb)

Additional file 15: **Table S6.** List of primers used for qRT-PCR analysis. (XLSX 8 kb)

## Abbreviations

AMP: Antimicrobial peptides
CLE: CLAVATA3 CLV3/ENDOSPERM SURROUNDING REGION ESR
CRP: Cysteine-Rich Peptides
EXPA6: Alpha-expansin
GAPDH: Glyceraldehyde-3-phosphate dehydrogenase
MeJA: Methyl jasmonic acid
MIC: Minimum inhibitory concentration
PDPs: Protein degradation patterns
PR10: Pathogenesis Related protein
PSK: Phytosulfokine
SA: Salicylic acid
UPS: Ubiquitin–proteasome system
XIC: Extracted ion chromatogram
