# Supplementary material for: Phytohormone treatment induces generation of cryptic peptides with antimicrobial activity in the Moss Physcomitrella patens
Source: BMC Plant Biol. 2019 Jan 7;19:9. doi: 10.1186/s12870-018-1611-z (PMC6322304; doi:10.1186/s12870-018-1611-z)
Supplement: Supplementary file 2 — Figure S2. The MRM chromatogram of eleven standard RT peptides added in cell and secretome samples. (PDF 613 kb) [file 12870_2018_1611_MOESM2_ESM.pdf]

Figure S2. The MRM chromatogram of standard RT peptides added in cell and secretome samples.

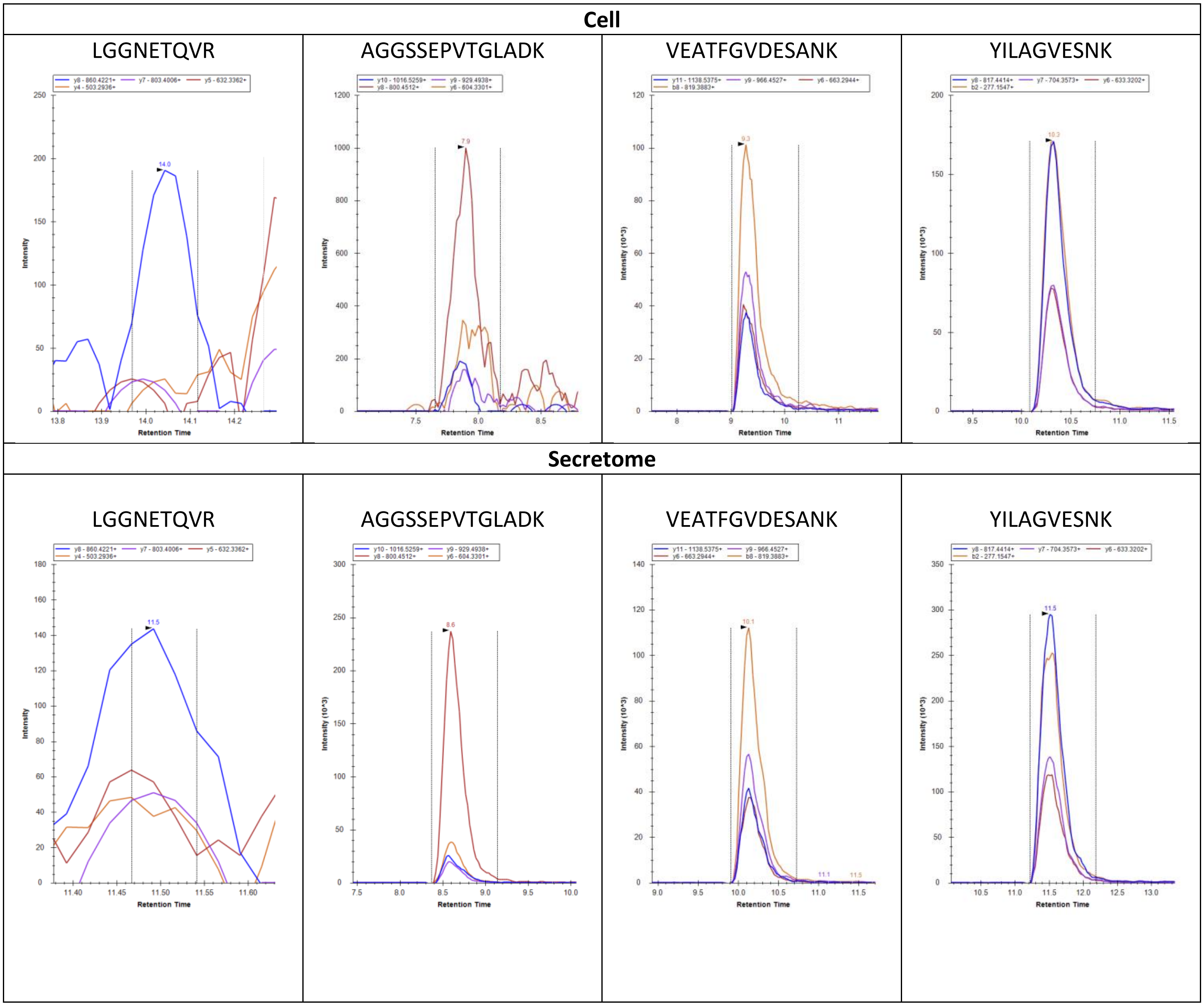

Cell

TPVISGGPYYER

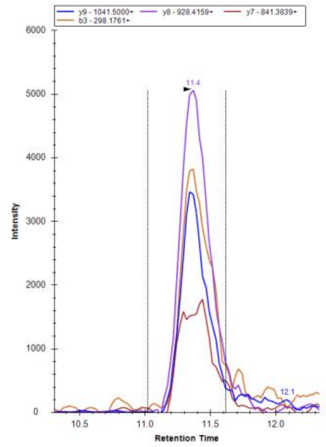

TPVITGAPYYER

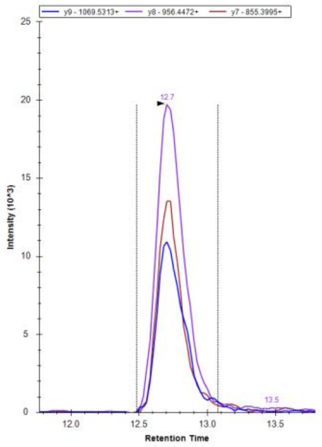

GDLDAASYYAPVR

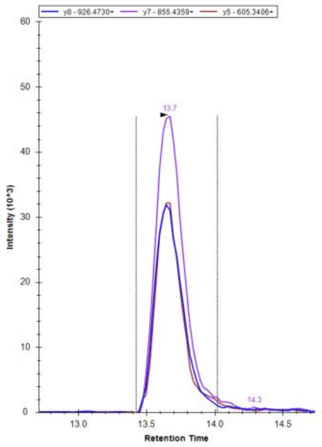

DAVTPADFSEWSK

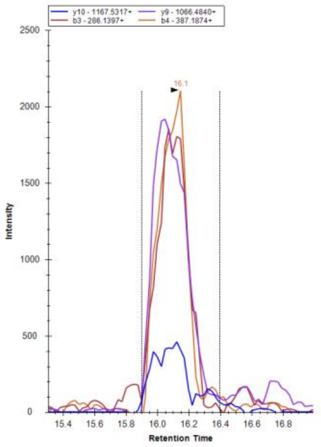

Secretome

TPVISGGPYYER

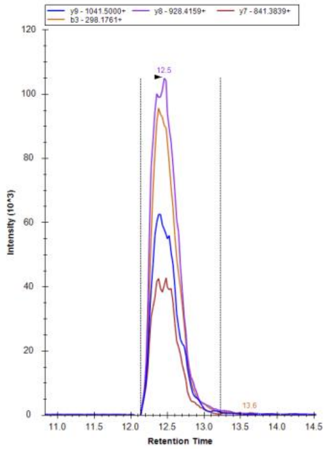

TPVITGAPYYER

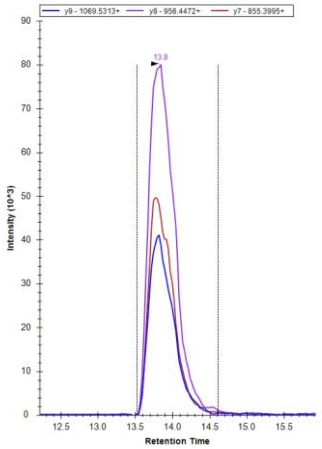

GDLDAASYYAPVR

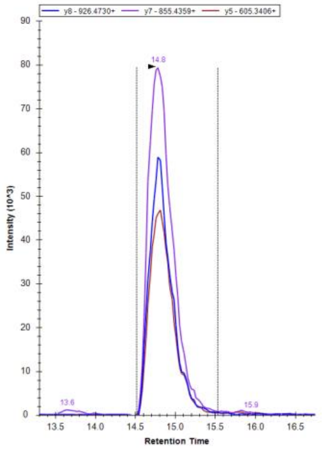

DAVTPADFSEWSK

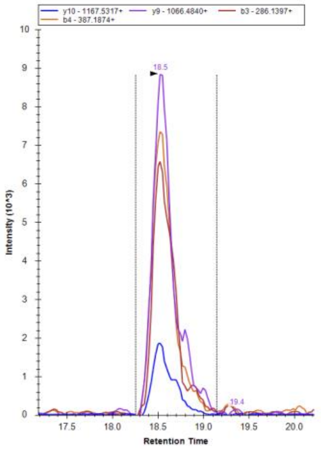

Cell		
TGFIIDPGGVIR	GTFIIDPAAIVR	FLLQFGAQGSPLFK
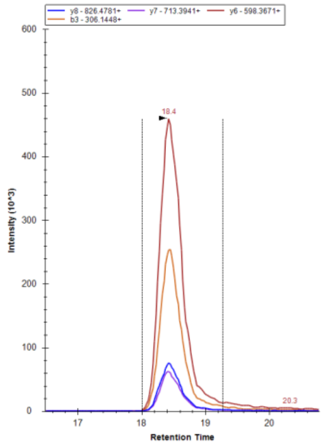	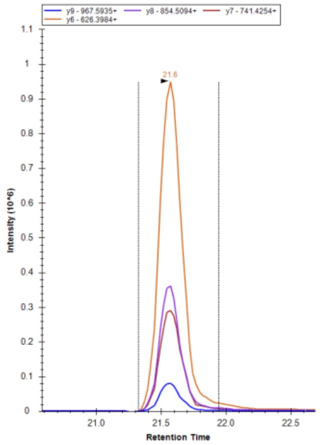	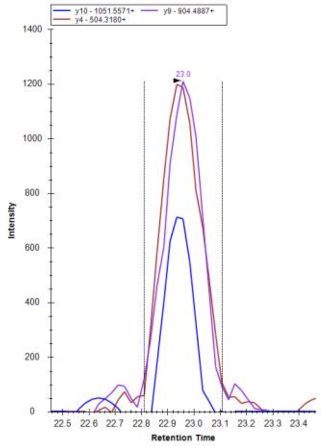

Secretome		
TGFIIDPGGVIR	GTFIIDPAAIVR	FLLQFGAQGSPLFK
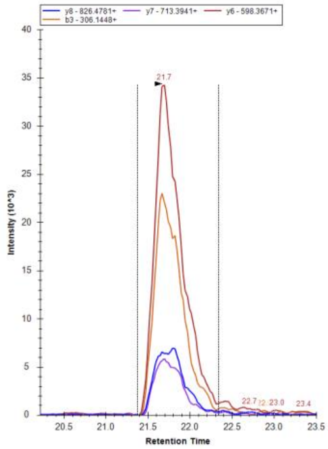	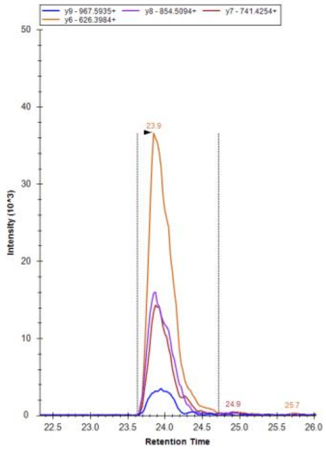	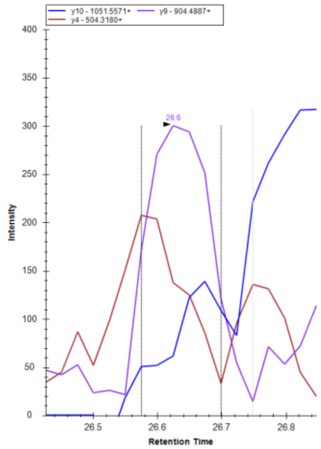
