# Supplementary material for: Phytohormone treatment induces generation of cryptic peptides with antimicrobial activity in the Moss Physcomitrella patens
Source: BMC Plant Biol. 2019 Jan 7;19:9. doi: 10.1186/s12870-018-1611-z (PMC6322304; doi:10.1186/s12870-018-1611-z)
Supplement: Supplementary file 5 — Figure S3. The distribution of the log2-transormed (log2_FC) peptide intensities based on Xtracted Ion Chromatogram (XIC) values. (PDF 129 kb) [file 12870_2018_1611_MOESM5_ESM.pdf]

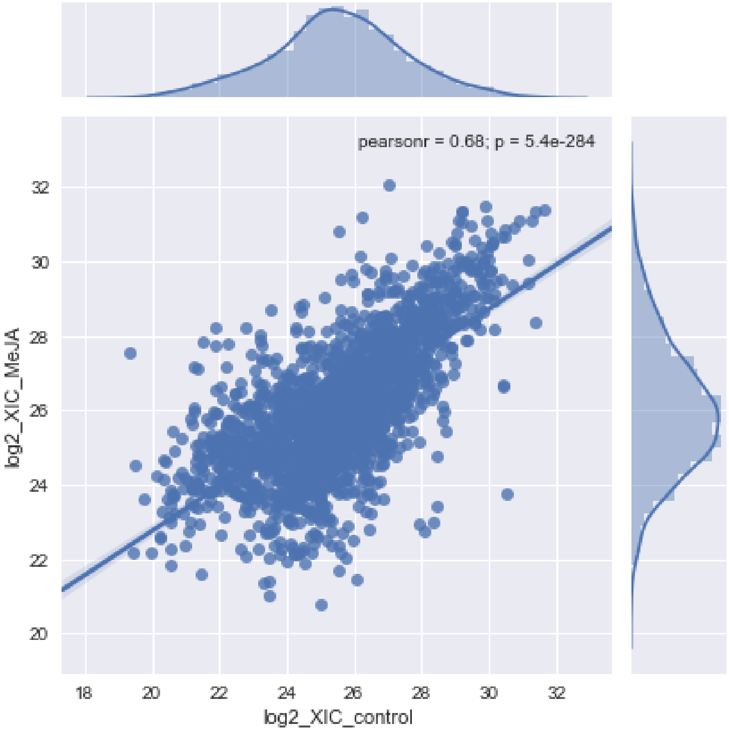

Figure S3. The distribution of the log_2-transormed (log2_FC) peptide intensities based on Xtracted Ion Chromatogram (XIC) values.
