# Supplementary material for: Phytohormone treatment induces generation of cryptic peptides with antimicrobial activity in the Moss Physcomitrella patens
Source: BMC Plant Biol. 2019 Jan 7;19:9. doi: 10.1186/s12870-018-1611-z (PMC6322304; doi:10.1186/s12870-018-1611-z)
Supplement: Supplementary file 6 — Figure S4. TreeMap showing GO enrichment analysis results for all protein precursors for cell peptides. (PDF 184 kb) [file 12870_2018_1611_MOESM6_ESM.pdf]

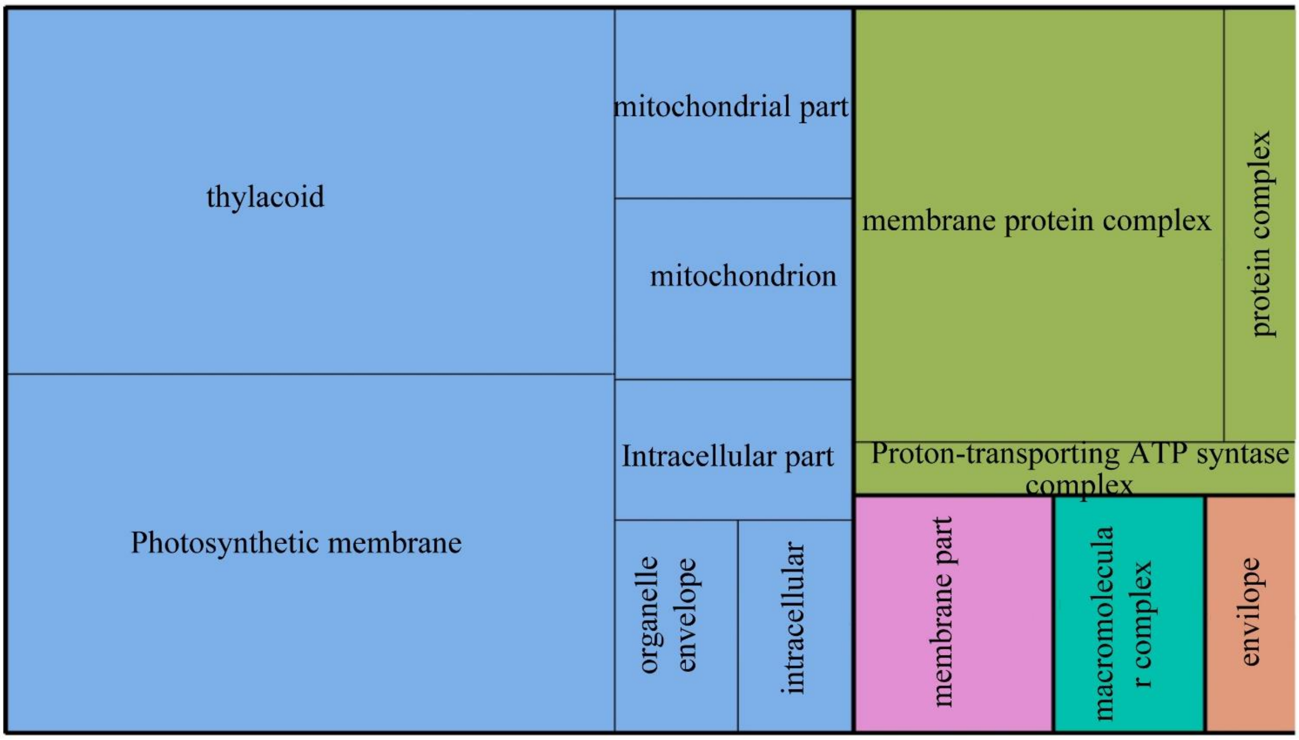

Figure S4. TreeMap showing GO enrichment analysis results for all protein precursors for cell peptides.
