# Supplementary material for: Phytohormone treatment induces generation of cryptic peptides with antimicrobial activity in the Moss Physcomitrella patens
Source: BMC Plant Biol. 2019 Jan 7;19:9. doi: 10.1186/s12870-018-1611-z (PMC6322304; doi:10.1186/s12870-018-1611-z)
Supplement: Supplementary file 8 — Figure S5. The barplot shows optical density of E. coli and B. subtilis cultures after 24-h incubation with secretome samples treated with different concentration of MeJA. The bars (M ± SD) represent the results of three independent experiments performed in triplicate. (PDF 220 kb) [file 12870_2018_1611_MOESM8_ESM.pdf]

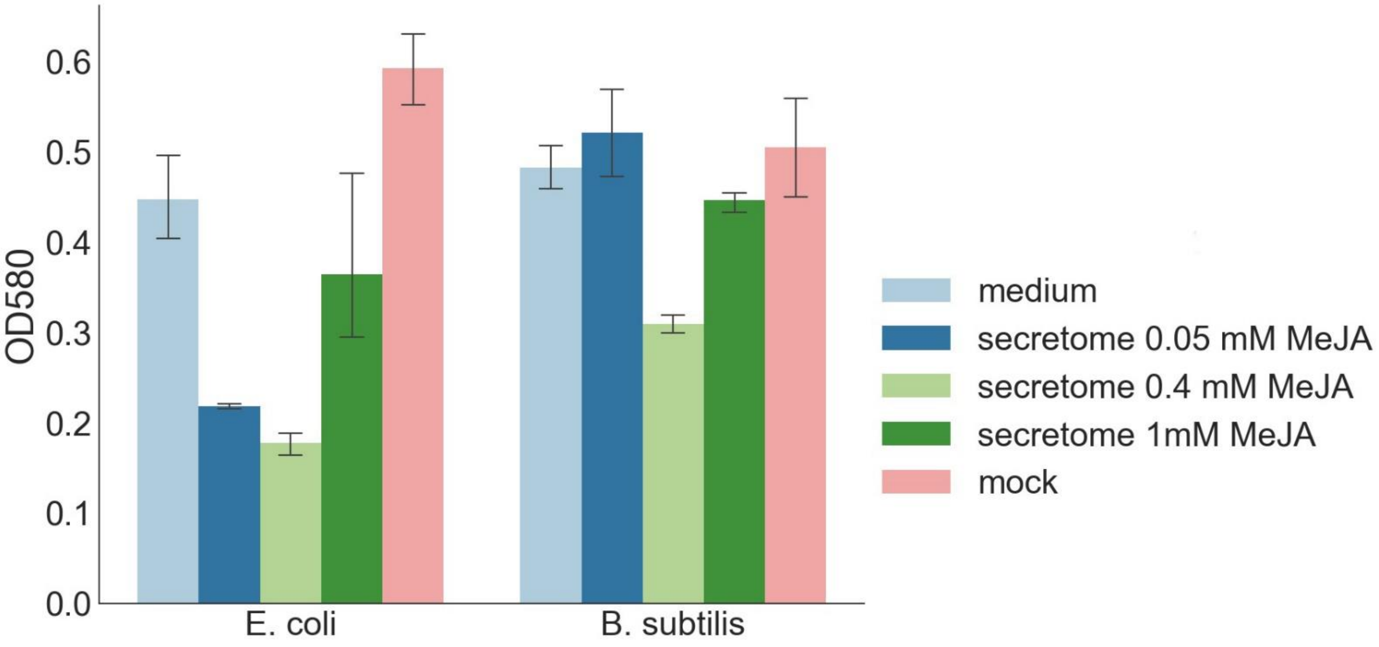

Figure S5. The barplot shows optical density of *E. coli* and *B. subtilis* cultures after 24-h incubation with secretome samples treated with different concentration of MeJA. The bars (M ± SD) represent the results of three independent experiments performed in triplicate.
