# Supplementary material for: Phytohormone treatment induces generation of cryptic peptides with antimicrobial activity in the Moss Physcomitrella patens
Source: BMC Plant Biol. 2019 Jan 7;19:9. doi: 10.1186/s12870-018-1611-z (PMC6322304; doi:10.1186/s12870-018-1611-z)
Supplement: Supplementary file 9 — Figure S6. The barplot shows optical density of E. coli and B. subtilis cultures after 24-h incubation with secretome samples. Secretome+inhibitor+MeJA – secretomes of moss protonema treated with 400 μM MeJA and the protease inhibitor cocktail; secretome+MeJA - secretomes of moss protonemata treated with the 400 μM MeJA; secretome+inhibitor - secretomes of moss protonema treated with the protease inhibitor cocktail. The bars (M ± SD) represent the results of three independent experiments performed in triplicate. (PDF 202 kb) [file 12870_2018_1611_MOESM9_ESM.pdf]

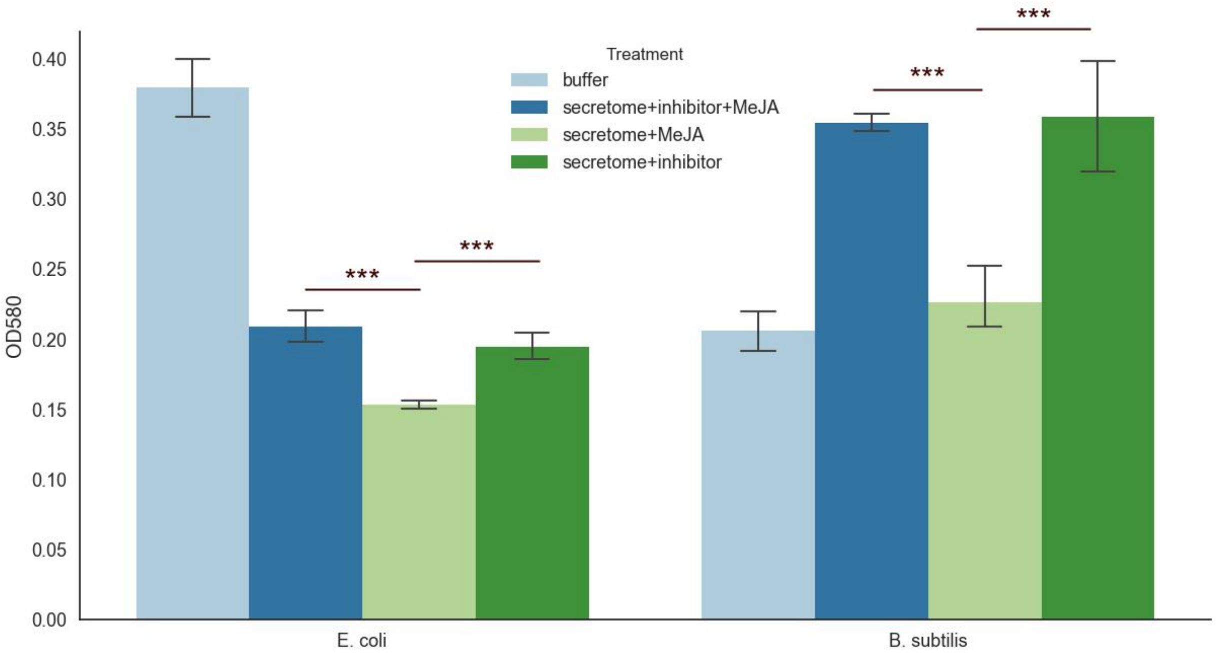

Figure S6. The barplot shows optical density of *E. coli* and *B. subtilis* cultures after 24-h incubation with secretome samples. **Secretome+inhibitor+MeJA** – secretomes of moss protonema treated with 400 μM MeJA and the protease inhibitor cocktail; **secretome+MeJA** - secretomes of moss protonemata treated with the 400 μM MeJA; **secretome+inhibitor** - secretomes of moss protonema treated with the protease inhibitor cocktail. The bars (M ± SD) represent the results of three independent experiments performed in triplicate.
