# Supplementary material for: Phytohormone treatment induces generation of cryptic peptides with antimicrobial activity in the Moss Physcomitrella patens
Source: BMC Plant Biol. 2019 Jan 7;19:9. doi: 10.1186/s12870-018-1611-z (PMC6322304; doi:10.1186/s12870-018-1611-z)
Supplement: Supplementary file 12 — Figure S7. Analysis of minimal inhibitory concentration (MIC) for the two peptides (Pep1- LVQIGTKIVGVGRNYAAH and Pep8- INIINAPLQGFKIA). Melittin was used as a positive control. The barplot shows optical density of B. subtilis and E. coli cultures after 24-h incubation with different peptide concentrations. (PDF 171 kb) [file 12870_2018_1611_MOESM12_ESM.pdf]

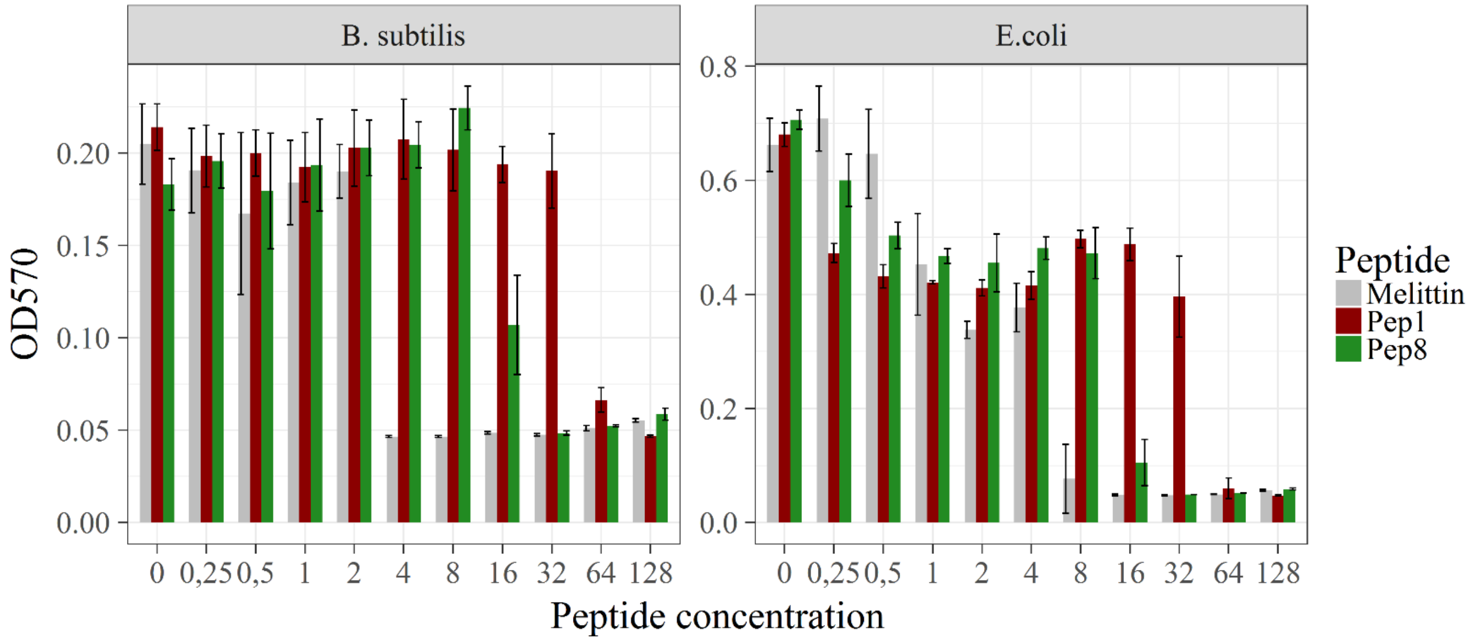

Figure S7. Analysis of minimal inhibitory concentration (MIC) for the two peptides (Pep1-LVQIGTKIVGVGRNYAAH and Pep8- INIINAPLQGFKIA). Melittin was used as a positive control. The barplot shows optical density of *B. subtilis* and *E. coli* cultures after 24-h incubation with different peptide concentrations.
