# Supplementary material for: Phytohormone treatment induces generation of cryptic peptides with antimicrobial activity in the Moss Physcomitrella patens
Source: BMC Plant Biol. 2019 Jan 7;19:9. doi: 10.1186/s12870-018-1611-z (PMC6322304; doi:10.1186/s12870-018-1611-z)
Supplement: Supplementary file 13 — Figure S8. (A) Results of quantitative polymerase chain reaction (qRT-PCR) for PAL and OPR genes after treatment of protonemata with 50μM, 100μM, 400μM and 1mM methyl jasmonate (MeJA). (B) Results of qRT-PCR for PAL and OPR genes after treatment of protonemata with 10μM, 100μM, 400μM and 1mM salicylic acid (SA); The normalized ratios and standard deviation of three independent triplicate experiments are shown. (PDF 182 kb) [file 12870_2018_1611_MOESM13_ESM.pdf]

A

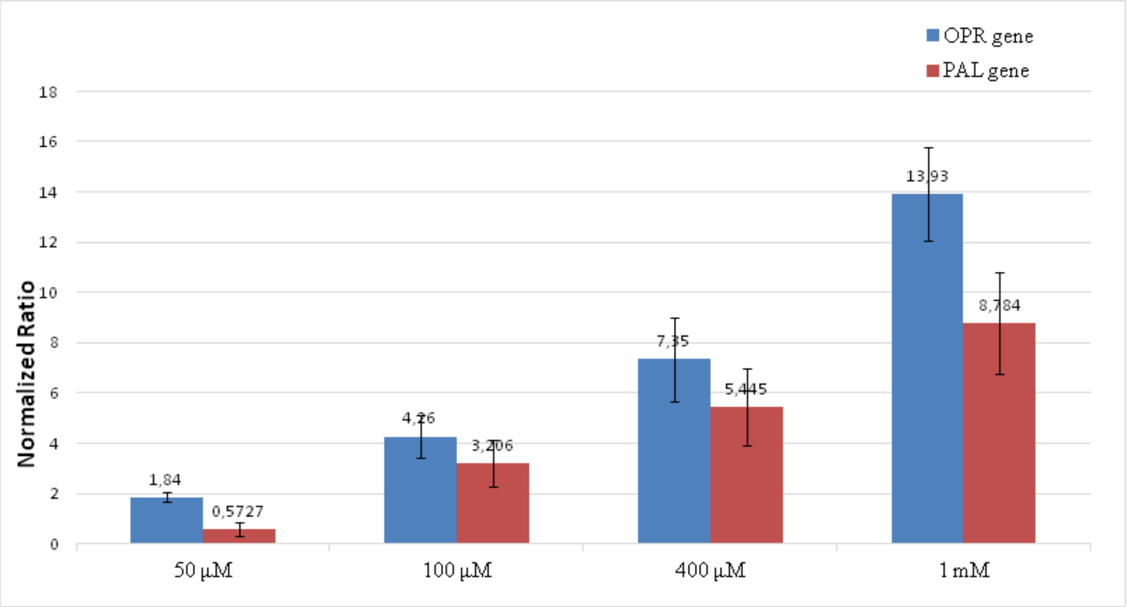

B

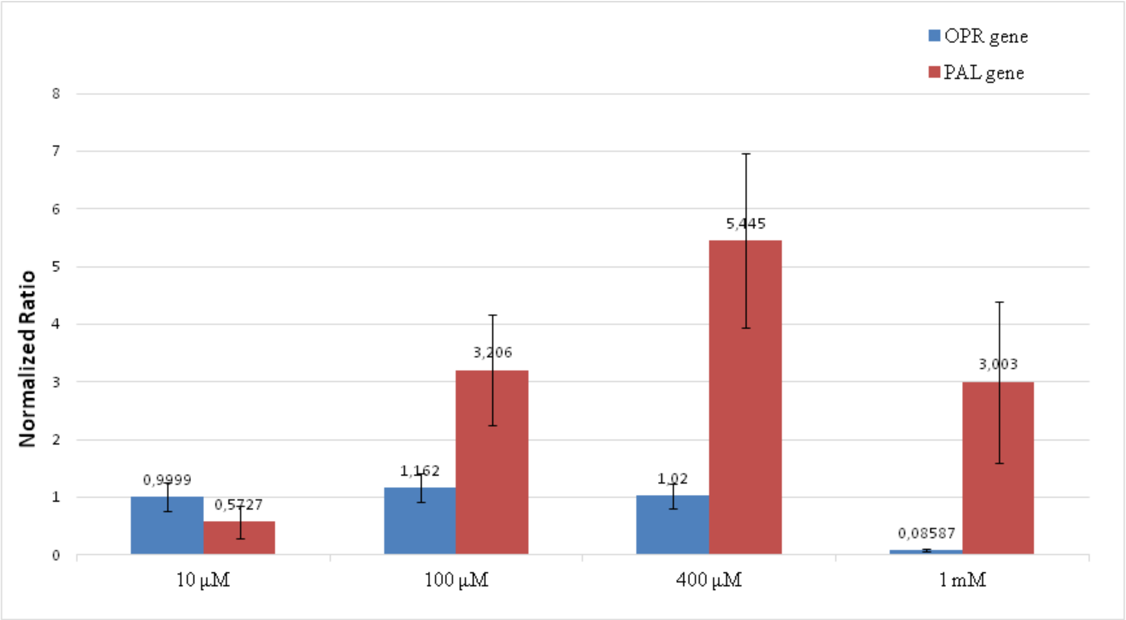

Figure S8. (**A**) Results of quantitative polymerase chain reaction (qRT-PCR) for PAL and OPR genes after treatment of protonemata with 50µM, 100µM, 400µM and 1mM methyl jasmonate (MeJA). (**B**) Results of qRT-PCR for PAL and OPR genes after treatment of protonemata with 10µM, 100µM, 400µM and 1mM salicylic acid (SA); The normalized ratios and standard deviation of three independent triplicate experiments are shown.
