# Supplementary material for: Phytohormone treatment induces generation of cryptic peptides with antimicrobial activity in the Moss Physcomitrella patens
Source: BMC Plant Biol. 2019 Jan 7;19:9. doi: 10.1186/s12870-018-1611-z (PMC6322304; doi:10.1186/s12870-018-1611-z)
Supplement: Supplementary file 14 — Figure S9. Venn diagram showing a comparison between protein precursors of our control dataset and proteome of P. patens bioreactor supernatants (Hoerstein et. al. 2018). (PDF 250 kb) [file 12870_2018_1611_MOESM14_ESM.pdf]

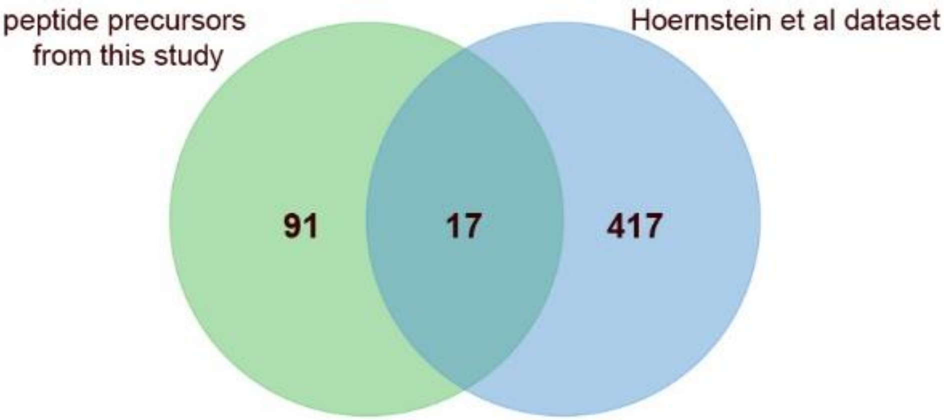


**Figure S9.** Venn diagram showing a comparison between protein precursors of our control secretome dataset and proteome of *P. patens* bioreactor supernatants (Hoerstein et. al. 2018).
